# The evolutionary ecology of the Lygaeidae

**DOI:** 10.1002/ece3.1093

**Published:** 2014-05-02

**Authors:** Emily R Burdfield-Steel, David M Shuker

**Affiliations:** Centre for Biological Diversity, School of Biology, University of St AndrewsHarold Mitchell Building, St Andrews, KY16 9TH, UK

**Keywords:** Ecology, entomology, evolution, life history, Lygaeidae, sexual selection

## Abstract

The Lygaeidae (*sensu lato*) are a highly successful family of true bugs found worldwide, yet many aspects of their ecology and evolution remain obscure or unknown. While a few species have attracted considerable attention as model species for the study of insect physiology, it is only relatively recently that biologists have begun to explore aspects of their behavior, life history evolution, and patterns of intra- and interspecific ecological interactions across more species. As a result though, a range of new phenotypes and opportunities for addressing current questions in evolutionary ecology has been uncovered. For example, researchers have revealed hitherto unexpectedly rich patterns of bacterial symbiosis, begun to explore the evolutionary function of the family's complex genitalia, and also found evidence of parthenogenesis. Here we review our current understanding of the biology and ecology of the group as a whole, focusing on several of the best-studied characteristics of the group, including aposematism (i.e., the evolution of warning coloration), chemical communication, sexual selection (especially, postcopulatory sexual selection), sexual conflict, and patterns of host-endosymbiont coevolution. Importantly, many of these aspects of lygaeid biology are likely to interact, offering new avenues for research, for instance into how the evolution of aposematism influences sexual selection. With the growing availability of genomic tools for previously “non-model” organisms, combined with the relative ease of keeping many of the polyphagous species in the laboratory, we argue that these bugs offer many opportunities for behavioral and evolutionary ecologists.

## Introduction

Insects of the family Lygaeidae (Insecta: Hemiptera: Heteroptera), commonly known as seed bugs, ground bugs or milkweed bugs, are found on every continent except Antarctica and are one of the three largest families (loosely defined; see below) within the Heteroptera. The Heteroptera is itself one of the most successful exopterygote suborders with approximately 40,000 species. Several Lygaeidae species are of economic importance due to their status as pests (Sweet 2000; Summers et al. 2010), and the ease with which some species can be maintained in the laboratory has meant that a number of species have been utilized as laboratory animals in a range of contexts (Feir 1974), particularly for studies of insect physiology (Feir 1974; Jin et al. 2010). As a result, a variety of information on their biology is available, albeit spread across a number of disciplines and journals. A great deal of this work is the result of research carried out by a small number of authors in the 1970s and 80s. However, a steady stream of literature on Lygaeidae has continued to be produced, particularly concerning their evolutionary ecology and sexual behavior. In contrast, *Oncopeltus fasciatus* has been the subject of considerable work on gene function and genetic control of development (Angelini and Kaufman 2004). Several key developmental genes have been cloned from the species (Angelini et al. 2005; Liu and Kaufman 2005; Erezyilmaz et al. 2006; Panfilio et al. 2006), and RNAi has been used to disrupt gene function (Angelini and Kaufman 2005). Given that work is also underway to sequence the *O. fasciatus* genome, and much of our basic knowledge of lygaeid biology has come from studies of this species (see Bonhag and Wick 1953 for example), it provides a valuable opportunity to explore the molecular mechanisms underpinning evolutionary ecology. Here we aim to bring this disparate literature together to make the family better known to evolutionary and behavioral ecologists, and also to highlight the potential to link reproductive physiology and ecology at both mechanistic and functional levels (e.g., Attisano et al. 2012). First, we place the Lygaeidae in its current (and admittedly poorly resolved) phylogenetic context, before considering aspects of the family's basic biology. We then explore in more detail the progress made in understanding the evolutionary ecology of this group of insects, considering the evolution of aposematism (warning coloration) and mimicry, population structure and patterns of migration, life history variation, mating system ecology, reproductive behavior and sexual selection. Importantly, chemical ecology appears to play a key part in many aspects of lygaeid biology and, as a result, we will keep returning to the role of chemical signals and chemical defence throughout this review.

## Phylogenetic Status and Basic Biology

**Figure 1:**
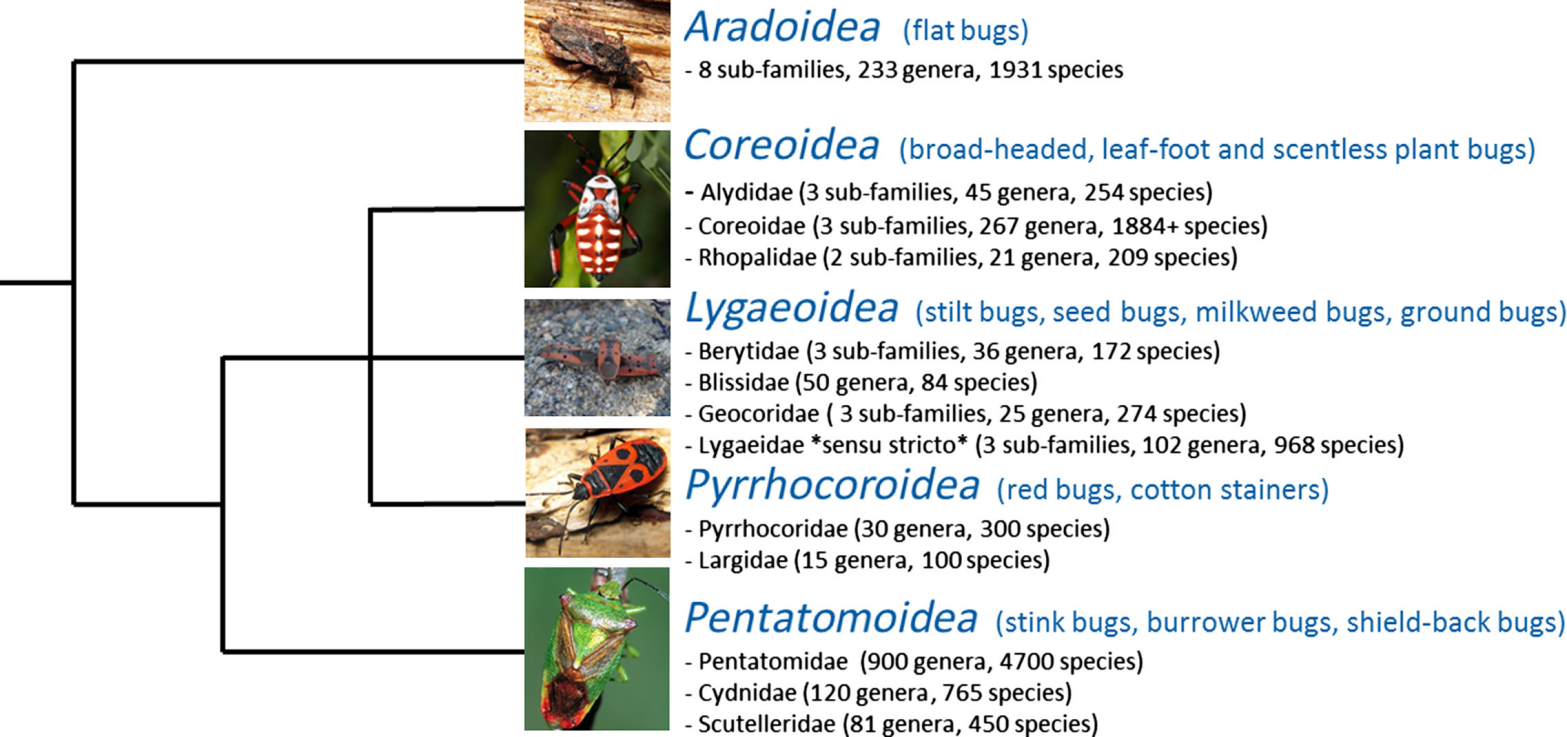
A schematic of the relationships among the infra-order Pentatomomorpha (after Li et al. 2005 and Henry 2009). Between 5 and 15 families are thought to comprise the Lygaeoidea, including the family Lygaeidae *sensu stricto*. Photo credits: The Tree of Life Web Project and David Shuker.

Recent studies have confirmed that the family Lygaeidae as traditionally defined is polyphyletic (Weirauch and Schuh 2011), and here we consider “lygaeids” in terms of the super-family Lygaeiodea. The lineages that currently make up the Lygaeiodea exist within the infra-order Pentatomomorpha (with putative sister-taxa Coreoidea and Pyrrhocoroidea, see Fig. 1), with the Cimicomorpha as the most likely sister taxon. We note, however, that further work on the families within the Lygaeoidea is required (Weirauch and Schuh 2011) as the suggested number of families varies from five to 15 depending on the authority (Henry 2009). Weirauch and Schuh recently proposed 11 families (including a family called Lygaeidae) (Weirauch and Schuh 2011). For simplicity, here we use the terms “Lygaeidae” or “lygaeid(s)” when discussing the bugs, not least as most of the biology has been done on species placed within the family Lygaeidae *sensu stricto* (e.g., the genera *Oncopeltus*, *Spilostethus,* and *Lygaeus*; see below). The poor resolution of the phylogeny across the Pentatomorpha is a clear constraint on comparative analyses that might wish to test evolutionary and ecological hypotheses, a constraint unfortunately shared with many other insect groups (e.g., Ross et al. 2012).

Lygaeids are typically small-to-medium-sized insects, ranging in size from approximately 1 to 12mm. While aposematism is widespread within the family, particularly within the subfamily Lygaeidae (Aldrich et al. 1997), the majority of species are cryptically colored (Schuh and Slater 1995). They are oval in shape and generally slender although notably some species of the subfamilies Cyminae and Pachygronthinae resemble the shape of the seeds they feed on (Schuh and Slater 1995). Most possess antennae made up of four segments, though some Lygaeidae have only three. They can be distinguished from their close relatives, the Coreidae, by the number of veins present on the forewing. Lygaeidae have five or fewer, while coreids have six of more. The Lygaeidae also closely resemble the Miridae but, unlike the mirids, they lack a distinctive cuneus and possess ocelli. The identification of key morphological features that characterize the Lygaeidae is problematic due to the polyphyletic nature of the family.

### Sexual systems

Chromosomal number in the Lygaeidae appears to vary considerably, and previous studies have reported male diploid chromosomal complements within the family ranging from six to 30 (Bressa et al. 2002; Souza et al. 2007). However, despite this extensive variation, chromosomal numbers of 2*n* = 14 and 2*n* = 16 are thought to be the most common (Kaur and Suman 2009). Sex determination within the Lygaeidae shows a similar diversity. While the majority (approximately 74% of Lygaeidae species studied as of 2007) have XY/XX sex determination, some species have X0/XX or multiple sex chromosomes (approximately 15% and 10%, respectively; Souza et al. 2007). The evolutionary biology of these changes is currently unknown. As discussed below, parthenogenetic populations of *Nysius groenlandicus* have also been recently discovered (Bocher and Nachman 2011).

### Oviposition

Female lygaeids generally lay eggs in clutches, which can range in size from 10 to over 100 eggs (Feir 1974; McLain 1991) and may lay many clutches in their lifetime. While parental care is widespread throughout the Heteroptera, with the most well-known example being the so-called “parent” bug *Elamucha grisea* (Tallamy and Schaefer 1997), no evidence has yet been found for parental care within the Lygaeidae beyond oviposition preferences. For example, some *Nysius* species are reported to attach their eggs individually to the underside of leaves (Tallamy and Schaefer 1997). While adults and nymphs of aposematic species can be found aggregating together, presumably to present a stronger aposematic signal (see below), such groupings are not necessarily formed of kin and therefore are not considered to be any form of parental care. Instead, eggs are laid in clutches, either in crevices in the ground or on host plants depending on the habitat of the species. Clutch size varies considerably within and between species and has been shown to be affected by temperature, food availability, photoperiod, population, female age and mating status (Table 1.).

**Table 1:** Recorded clutch sizes and lifetime egg production of several Lygaeidae species. Where these measures were unavailable other measures of egg production were substituted if available

Species	Number of eggs	Factors that influence egg production	References
*Lygaeus creticus*	Mean clutch size 20.7 ± 1.76 for once-mated females (at 29°C), lifetime egg production unknown	–	Burdfield-Steel (unpublished)
*Lygaeus equestris*	Clutch sizes range from 20 to 50 eggs. Lifetime egg production typically ranges from 300 to 500 eggs; however, this can be exceed this with some females producing up to 1000 eggs	Temperature, Population	Sillen-Tullberg (1981), Sillen-Tullberg and Solbreck (1990), Solbreck et al. (1989), Burdfield-Steel (unpublished)
*Lygaeus simulans*	Larger clutches exceed 60 eggs. Mean number of eggs laid per female in the laboratory was 150.2 after one successful mating and mean number of clutches was 5	Mating status	Tadler (1999)
*Neacoryphus bicrucis*	Females lay clutches of approximately 20 eggs almost daily The mean number of eggs produced per female over a 6 week period is approximately 143. Older females produced more eggs that younger ones	Female age	McLain (1991), McLain and Pratt (1999)
*Nysius huttoni*	200–600 eggs laid over the course of the females’ life. Dissected females contain 4–9 mature eggs	–	Yang and Wang (2004), Wang and Davis (2006)
*Oncopeltus cingulifer*	The average clutch size was 29 at 27°C and 17.3 at 25°C. Lifetime egg production was 222 at 25°C	Diet	Phelan and Frumhoff (1991), Root and Chaplin (1976)
*Oncopeltus fasciatus*	Typical clutch size is approximately 30 eggs though this appears to be highly variable (recorded clutch sizes range from 5 to more than 50). Lifetime egg production is similarly variable with some studies reporting between 200 and 2000 eggs produced by a female over her lifespan	Temperature, Population, Photoperiod	Sauer and Feir (1973), Dingle (1974), Feir (1974), Baldwin and Dingle (1986), Groeters and Dingle (1987), Dingle, Evans and Palmer (1988), Groeters and Dingle (1988), Eslie (1990), Phelan and Frumhoff (1991), Attisano et al. (2012), Burdfield-Steel (unpublished)
*Oncopeltus unifasciatellus*	Average clutch size 26.1 eggs (at 25°C). Lifetime egg production approximately 749 eggs	Diet	Root and Chaplin (1976)
*Ozophora baranowskii*	Females had an oviposition rate of 4–6 eggs per day	–	Rodríguez (1998)
*Spilostethus pandurus*	Mean clutch size 41.9 ± 3.16 for one-mated females (at 29°C)	–	Burdfield-Steel (unpublished)
*Togo hemipterus*	Females lay 137.6 ± 10.9 (mean ± SE) eggs throughout their lifetime at a rate of 3.4 ± 1.1 eggs per day for 45.8 ± 3.6 days	–	Himuro and Fujisaki (2010)

Nymphs typically live in similar environments to their parents (Schuh and Slater 1995) and are often gregarious (Aller and Caldwell 1979). Importantly, sibling cannibalism is known to occur in Lygaeidae (Sweet 2000), particularly during and shortly after hatching (pers obs.; Solbreck and Sillen-Tullberg 1990). Indeed, the seemingly high frequencies of infertile eggs observed in laboratory populations of several lygaeid species suggests a potential role of provisioning for these eggs (so-called “trophic” or “nurse” eggs) as newly hatched nymphs will often eat unhatched eggs (pers obs.; Root and Chaplin 1976; Solbreck and Sillen-Tullberg 1990). Indeed, newly hatched *Spilostethus pandurus* nymphs prefer to attack unfertilized eggs over unhatched fertilized eggs, and consumption of a single egg by a newly hatched nymph doubled their survival time compared with a starved nymph (Anderson and Solbreck 1992). This may be particularly important as *S. pandurus* frequently lay their eggs some distance from food sources (Anderson and Solbreck 1992). In a recent review of trophic egg theory and experiment, Perry and Roitberg (2006) outline the key hypotheses and what experiments would be needed to test them. For instance, in many cases of apparent trophic egg production across insects, it is currently not clear whether unfertilized eggs are deliberately produced or are the result of other factors such as sperm limitation (Perry and Roitberg 2006). It is also not clear whether trophic eggs, if deliberate, are produced as a form of offspring provisioning or are instead produced to limit sibling cannibalism. In order for this to be the case, we would expect these “trophic” eggs to be less costly to produce than a viable egg. However, to the authors’ knowledge, there are currently no studies that compare the structure and chemical composition of unhatched eggs and viable ones in the Lygaeidae. Interestingly, it is currently also unknown if the observed levels of sibling cannibalism reflect parent–offspring conflict. It has been suggested that females could manipulate hatching synchrony to reduce sibling cannibalism (Schausberger and Hoffmann 2008) as early hatching nymphs will attack unhatched eggs. However, theory developed in birds suggests that females may also manipulate clutch size in order to regulate sibling conflict (Nilsson 1995). Lygaeidae may provide an interesting system for the study of parent–offspring conflicts in the form of sibling competition as their promiscuous mating system may frequently result in clutches of mixed parentage (Economopoulos and Gordon 1972; Sillen-Tullberg 1981; Wang and Davis 2006). If the average relatedness of a lygaeid nymph to its clutch mates is less than that of the mother to her offspring, then the potential for such conflicts may be greater than in typically monogamous species. The scope for the study of parent–offspring and offspring–offspring conflict is clear, and numerous lygaeids may be suitable study systems.

### Development

As with all hemimetabolous insects, the Lygaeidae do not undergo complete metamorphosis during their life cycle, but instead typically have five wingless nymphal instars before becoming reproductive, and typically winged, adults (Slater and Gagne 1973; Cárdenas et al. 2001). However, flexibility in the number of larval stages has been observed in the New Zealand species *Nysius huttoni*. In this species, the number of larval instars ranges from three to seven, though individuals with five instars still form the majority. In the laboratory, variation in the number of instars is affected by both temperature and photoperiod, with lower numbers of instars more frequent at lower temperatures (Wei 2010). This suggests that flexibility in the number of larval instars may aid survival under changing environmental conditions (Wei 2010). *N. huttoni* normally have three generations per year in their natural range and overwinter as adults; therefore, a shorter nymphal stage could potentially be beneficial to enable individuals to reach adulthood during poor, shorter summers. So far, this is the only species found to show this flexibility in nymphal instars within the Lygaeidae; however, it is well documented in other insect groups (Esperk et al. 2007) and may well be far more common. Evidence for another form of developmental variation has been found in *Oncopeltus fasciatus* in which maternal effects influence size and development time, particularly with respect to the age of the mother (Phelan and Frumhoff 1991). This has also been linked to the seasonal variation experienced by some populations and has been suggested as a mechanism by which mothers can maximize the survival potential of their offspring as the offspring of older mothers develop faster (Groeters and Dingle 1987). The link between nymph size and speed of development is strengthened by evidence that the timing of adult molt is determined by nymph weight, and delayed molting under low-nutritional conditions may be a result of failure to reach the critical weight to trigger the required hormonal changes (Blakley and Goodner 1978).

The pattern of development can also have important consequences for the evolution of life histories more generally. For instance, tropical populations of *Oncopeltus fasciatus* and the congener *O*. *cingulifer* experience naturally occurring variation in host plant quality, which influences the growth and development of nymphs. On poorer quality food (i.e., vegetative plant parts rather than nutrient-rich seeds), nymphs take longer to reach a given size, leading to a lower probability of surviving to eclosion. On the other hand, insects that eclose into adults at a larger body size have a better chance of survival under conditions of food stress and perhaps are better able to disperse away to better quality food patches (for instance with flowering host plants; Blakley 1981). This means that critical size at metamorphosis may be under conflicting selection pressures in juveniles and adults. Moreover, if these size–development–survival relationships differ between the sexes, we may also expect sexually antagonistic selection over development under nutritional stress.

### Diapause

A number of species in temperate areas, including *Lygaeus equestris* and the closely related species *L. simulans*, show reproductive diapause (i.e., diapause as adults) and migratory capabilities triggered by temperature and photoperiod (Solbreck 1979; Dingle et al. 1980a; Solbreck and Sillen-Tullberg 1981). These are thought to be adaptations to allow them to avoid or survive low temperatures during the winter months, as well as to migrate to follow seasonal patterns in host plant abundance (Dingle et al. 1980a; see also Attisano et al. 2013). Migration in *L. equestris* involves moving to overwintering sites, such as buildings or natural rock formations, rather than south to warmer climates (Solbreck 1976; Solbreck et al. 1989; Sillen-Tullberg and Solbreck 1990). Ovary development in the adult females does not start until after the spring migration flights from these overwintering areas to their breeding sites. As only adults can make the flights, typically the populations in northern Europe are univoltine, as only one generation can be produced per year. However, occasionally, during particularly warm years, two generations have been recorded, and the degree of multi-voltinism increases with decreasing latitude. Additionally, in *L. equestris,* copulation may determine the timing of diapause, as reproductively active females have been shown to be less likely to enter diapause (Sillen-Tullberg 1984).

### Feeding biology and economic impact

Turning to feeding, as one of the common names suggests, many, though not all, species of Lygaeidae feed on seeds (Schuh and Slater 1995). Like all Hemiptera, they have piercing–sucking mouth parts and feed through a flexible feeding tube called a proboscis. The proboscis, or rostrum, is made up of the mandibles and maxillae modified to form needle-like stylets lying within a grooved labium. Within this structure are two canals, one to deliver saliva and the other to take up food (Gullan and Cranston 2005). Feeding methods can be divided into two “types” or manners of feeding: “stylet-sheath” feeders and “lacerate-flush” feeders (Schuh and Slater 1995). In stylet-sheath feeders, the salivary glands produce a so-called “feeding cone” that connects the apex of the labium to the feeding substrate. In addition, a salivary sheath may be produced that lines the puncture in the seed or plant through which the stylets are inserted (Schuh and Slater 1995; Sweet 2000). Lacerate-flush feeders on the other hand use the barbed apical portion of the proboscis to macerate the internal tissues of the host, be it plant or animal (Schuh and Slater 1995). The tissues are then mixed with saliva, often containing digestive enzymes, and the resulting liquid is then sucked up through the food canal (Gullan and Cranston 2005). Both feeding mechanisms require the production of saliva, and at least in the laboratory, access to water is crucial for successful rearing.

The majority of Lygaeidae are lacerate-flush feeders, the method commonly used by species that feed on portions of the plant that give a high nutrient return, such as seeds. However, an unnamed lygaeid species has been observed to secrete a feeding cone when using the lacerate-flush method and produce a stylet-sheath when feeding on plant sap (Schuh and Slater 1995). As sap feeding is widespread throughout the Lygaeidae, and many lygaeids do feed on sap in addition to seeds, it is likely that this behavior may well apply to other species (Schuh and Slater 1995). Moreover, the families Blissidae, Malcidae, and Colobathristidae are predominantly sap feeders. The Blissidae, also known as chinch bugs, are arguably the most economically important group of lygaeids due to their status as pests (Samuels et al. 2002). For example, they are known to attack grasses including grain crops, sugar grasses and grasses used for lawns and playing fields (Sweet 2000). Another lygaeid known to attack crops is the North American pest species, the false chinch bug, *Nysius raphanus*. *N. raphanus* is multi-voltine, with adults overwintering on the ground underneath debris or rubbish (Demirel and Cranshaw 2006). Populations can expand rapidly during favorable conditions, and both adults and nymphs have been reported attacking a great variety of plants including tomatoes (Summers et al. 2010), cotton, tobacco, grapes, and turf (Sweet 2000), especially during the late spring when their preferred host plants begin to desiccate (Summers et al. 2010). The bugs aggregate in large groups on the plants and cause wilting, browning of the leaves, and deformation of any developing fruit (Sweet 2000). Interestingly, species of the family *Geocoris* have been reported as predators of *Nysius spp*. (Sweet 2000), suggesting they may be potential biological control agents of *Nysius raphanus*. Another notable lygaeid pest is *Spilostethus pandurus,* which has been reported to attack thirty-three crops species worldwide (Sweet 2000). As this species is highly polyphagous, it can switch from its preferred host plant, usually from the subfamily *Asclepiadaceae,* to various crops. Despite being predominantly a seed eater, the presence of *S. pandurus* on crops has been shown to reduce the number of fruit developing as well as damage the body of the plant due to the sucking of sap from the leaves, stalks, and flowers by both adults and nymphs. In severe cases, attacks by *S. pandurus* can cause plants to wither completely. In addition, the species has been implicated in the spread of the fungus *Nematospora corgli* which causes yellow-spot disease and is thought to transfer the fungus via its mouth parts (Sweet 2000). Finally, *L. equestris* have been reported as pests of sunflower seeds (Horvath and Frank 2002; Horváth et al. 2004).

It is unclear what proportion of lygaeid species are food plant specialists as opposed to generalists, as reliable data on habitat and feeding habits are available only for a few species (Ralph 1976; Solbreck et al. 1989). Several species, such as *Oncopeltus fasciatus*, have been classified as “milkweed-specific” as they are restricted to milkweeds and other plants of the subfamily Asclepiadaceae (Wheeler 1983). However, other species such as *Lygaeus equestris* (Kugelberg 1973, 1974)*, L. kalmii* (Wheeler 1983)*, Spilostethus pandurus* (Sweet 2000; Elbanna et al. 2009), and *Neacoryphus bicrucis* (Solbreck and Pehrson 1979) have been found to feed on numerous plant families, even though asclepiads remain their preferred hosts (Wheeler 1983). This host flexibility may account for many species’ sporadic role as pests. *S. pandurus* in particular often damages crops when its usual host plants are reduced by adverse weather or rendered insufficient by sudden population growth (see above). Laukkanen et al. (2013) recently looked for evidence for genetic trade-offs between the ability to grow on one or several host plants that might favor the specialization of herbivores, using *L. equestris* and four plant species, including the preferred host plant *Vincetoxicum hirundinaria*. They found no evidence for trade-offs in survival, development time, or mass, suggesting that trade-offs do not affect the ability of *L. equestris* to adapt to novel food plants.

However, there is some evidence for a trade-off between survival and reproduction in *O. fasciatus* when feeding on novel food plants. Females show evidence of adaptive oosorption in response to a novel diet, thus conserving resources normally invested in eggs during periods of food stress (Moore and Attisano 2011). This ability to reabsorb eggs may represent an adaptation that allows the short term utilization of a greater variety of host plants, allowing females to survive periods where their preferred food plants are scarce. During these periods, females may invest less in reproduction so as to increase survival. Thus, lygaeids may offer an interesting set of model systems for the study of the evolution of life histories under nutritional stress and in response to novel diet.

Despite being primarily plant feeders, the Lygaeiodea contains one family, the Geocoridae, in which carnivory has become the most common feeding habit. Not surprisingly, some geocorids are therefore potentially important biocontrol agents (e.g., in the genus *Geocoris* (Carstens et al. 2008; Tillman 2011; Lundgren 2011), and their ecological interactions have received quite a lot of attention, particularly in the context of intraguild predation (Polis et al. 1989; Müller and Brodeur 2002). Perhaps, more surprisingly, a small rhyparochromine lygaeid tribe, the Cleradini, has evolved to feed on the blood of small rodents (Harrington 1983), although a study on *Clerada apicicornis* suggested that this species, whilet facultatively hematophagous, prefers to feed on other insects, including the blood-feeding reduviid bug *Rhodnius prolixus* (Torres et al. 2000). The observations of Torres et al. mean that the records of hematophagy in *C*. *apicicornis* obtained from museum specimens by Harrington (Harrington 1990) might represent vertebrate blood meals obtained by feeing on other blood-feeders (so-called “cleptohaematophagy”) rather than from vertebrate hosts directly.

**Figure 2:**
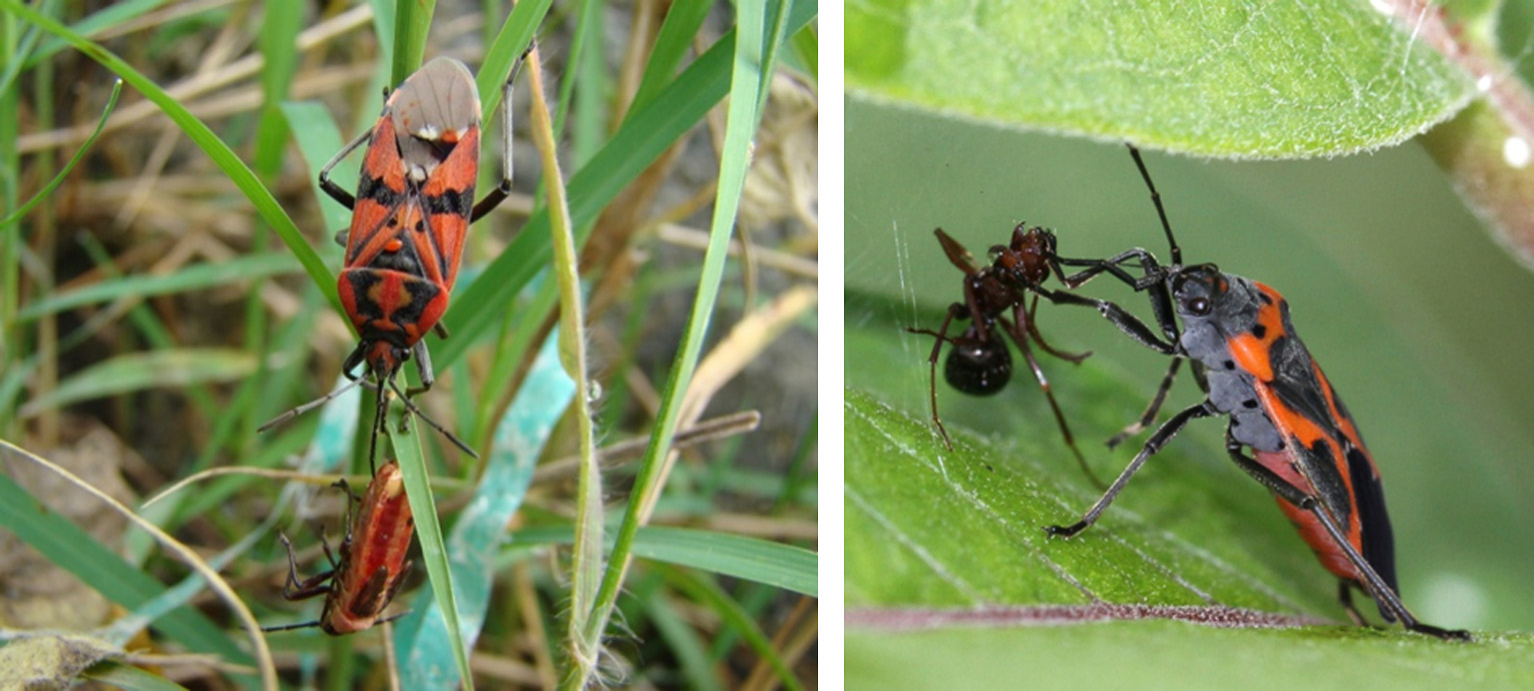
Left - An adult *Spilostethus pandurus* feeding on a *Lygaeus creticus* nymph. Sicily. Right – A *Lygaeus kalmii* feeding on an ant. Photo credits: (left to right) David Shuker, Mary Holland.

Generally, it appears that strict carnivory or phytophagy will be uncommon, with certain geocorids having been shown to be omnivorous, for example *Geocoris uliginosus* (Carstens et al. 2008). In addition, several otherwise phytophagous species having been observed engaging in opportunistic predation (e.g., Sweet 1979, 2000; Fig. 2); indeed, this habit may well be common. Sweet (1979) observed that plants are typically low in protein and that secondary carnivory may well have evolved in a number of lygaeid species to provide an additional source of protein. Likewise, as we have already seen, egg cannibalism seems to be common among newly hatched nymphs and may either represent offspring provisioning by the mother via the production of trophic eggs, or simple cannibalism of eggs that develop more slowly or fail to hatch (Perry and Roitberg 2006). As previously discussed, such cannibalism could select for mothers to promote hatching synchrony, and for offspring to try and hatch first, leading to within- and between-generational conflict over time spent as an embryo. Eggs are also cannibalized by later-instar nymphs and also adults (Solbreck and Sillen-Tullberg 1990) which is an important consideration for laboratory studies. Currently, there are only limited data testing kin discrimination of eggs/nymphs in terms of cannibalism, and much remains to be discovered.

### Endosymbionts

Interestingly, from across the insects, there is the hint of an apparent syndrome of aposematism, sibling cannibalism, and the presence of male-killing endosymbionts (Majerus 2003). To date, only one population of *Spilostethus hospes* has been identified as harboring an (unidentified) male-killer bacterium (Groeters 1996), with circumstantial evidence from a population of *Oncopeltus fasciatus* of a similar kind of sex ratio distorter (Leslie 1984). However, we predict that such sex ratio distorting endosymbionts will be more common in lygaeids than currently appreciated due to the habit of sibling cannibalism. In terms of symbiotic bacteria more generally, as with many of their Homoptera cousins (such as aphids, whiteflies and scale insects), many Heteroptera with restricted diets (such as blood-feeding cimicid bugs or sap feeding pentatomids) have bacterial symbionts, which are thought to facilitate production of otherwise limiting micro-nutrients. However, in Heteroptera, these tend to be extracellular symbionts, housed in midgut sacs, crypts, or tubules, rather than within cells (see Kuechler et al. 2010; Matsuura et al. 2012). This extracellular habit requires an alternative suite of adaptations for cross-generational transfer of these useful bacteria (such as *Gammaproteobacteria*), such as mother–offspring transfer via so-called “egg smearing,” or gaining the bacteria from the environment (Kuechler et al. 2012). Recently, however, exceptions to this pattern have been found in a burst of exciting new papers.

First, the lygaeid *Kleidocerys resedae*, or birch catkin bug, has been found to have an obligate endosymbiotic proteobacterium which lives in a specialized endocellular mycetome structure (Kuechler et al. 2010). This species also has alpha-proteobacteria of the genera *Wolbachia* and *Rickettsia*. Second, the bulrush bug *Chilacis typhae* (Lygaeidae, subfamily Artheneinae) hosts an obligate intracellular gamma-proteobacterium in a set of enlarged midgut cells, arranged in a circular fashion around the midgut (called a “mycetocytic belt” by the authors: Kuechler et al. 2011). These authors suggest that this structure might be an intermediate structure between the midgut crypts or sacs of other Lygaeioids, Pentatomoids and Coreiods, and the more specialized bacteriome/mycetome of *Kleidocerys* (Kuechler et al. 2011).

Third, four species of *Nysius* (*Nysius plebeius*, *N*. *expressus,* and two species not identified to species level) have been found to harbor the primary gamma-protobacterial endosymbiont *Schneideria nysicola* in a pair of bacteriome structures (Matsuura et al. 2012). These authors also identified *Wolbachia* and a novel alpha-proteobacterium in these species, finding the latter in five other species across the super-family as well. The gammaproteobacterium in *Nysius* does not form a clade with the bacterium found in *Kleidocerys*, suggesting an independent evolutionary origin of this symbiosis, with strict host-symbiont coevolution within the four *Nysius* species (Matsuura et al. 2012).

Fourth, five species from the Blissidae (*Ischnodemus sabuleti*) and Lygaeidae (*Arocatus longiceps*, *Belonochilus numenius*, *Orsillus depressus*, and *Ortholomus punctipennis*) have also been confirmed to host gamma-proteobacteria endosymbionts in paired bacteriome structures (Kuechler et al. 2012). The phylogenetically diverse symbionts and the anatomical characteristics and location of the bacteriomes varied across the species, suggesting either independent evolutionary origins for the symbioses or (perhaps) some coevolution among hosts and symbionts. In addition to these findings, *Wolbachia* has also been recorded from two species of *Nysius* (not identified to species level: Weeks et al. 2003) and from a range of Heteroptera including 14 out of 24 species of Japanese Lygaeiodea (comprising species from the Lygaeidae, Malcidae, and Berytidae; (Kikuchi and Fukatsu 2003). All told, these surveys suggest that more symbionts, including obligate endosymbionts with specialized host structures, probably await discovery. Given the possible ecological and evolutionary consequences of these symbionts (Moran et al. 2008), and perhaps the opportunity to study a range of colonization events and a diversity of host specializations (from simple crypts to dedicated host tissues) in the same super-family, there should be much interest in exploring this aspect of lygaeid biology further.

### Social interactions

**Figure 3:**
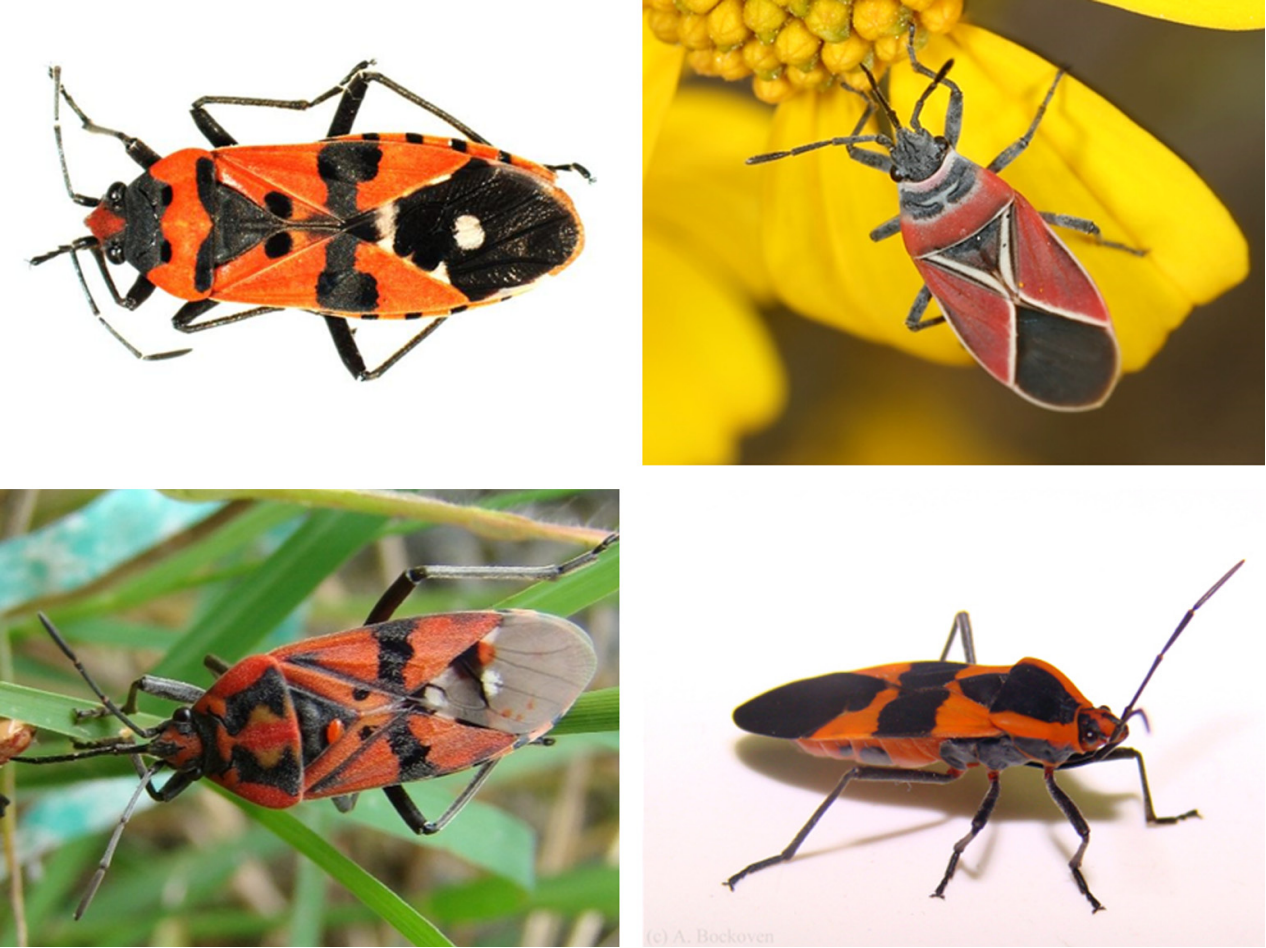
Four extensively studies species of Lygaeidae. Top right *Neacoryphus bicrucis* (photo courtesy of Jillian Cowles), bottom right *Oncopeltus fasciatus* (photo courtesy of Alison Bockoven), bottom left *Spilostethus pandurus* (David Shuker) and top left *Lygaeus equestris* (Liam Dougherty).

**Figure 4:**
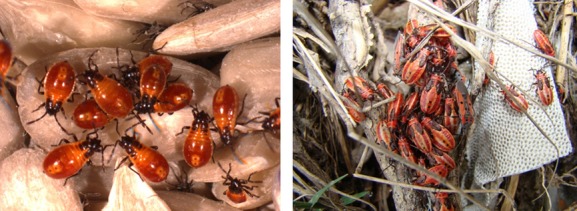
Right – Laboratory raised second instar nymphs of the species *Spilostethus pandurus* showing characteristic black and red coloration. Left – an aggregation of late-instar *Lygaeus creticus* nymphs and adults. Sicily. Photo credits: Emily Burdfield-Steel.

In addition to intraguild predation, a variety of social behaviors and interactions are apparent across the Lygaeidae. Individuals of many species, including *Oncopeltus fasciatus*, *Spilostethus pandurus* and several species of the genus *Lygaeus* aggregate as nymphs (Aller and Caldwell 1979) as well as adults (Root and Chaplin 1976; Fig. 3). These aggregations may occur during nymph development, when adults may also be present (Fig 4), or during periods of hibernation (e.g., hibernating masses of *Lygaeus equestris*: (Solbreck and Sillen-Tullberg 1990). Rather than try and characterize these interactions as “sub-social” or “communal,” we follow Costa (2006) and consider all these behaviors as “social.” Otherwise, adults are typically solitary barring sexual interactions, which are considered in more detail below, although food plants may often host several individuals of both sexes which may stay on the same plant for several days (e.g., *Spilostethus pandurus* on the milkweed *Gomphocarpus sinaicus*: (Elbanna 2004; Elbanna et al. 2009). While nymphal aggregation may simply be the result of kin-aggregation in *Oncopeltus fasciatus*, this social behavior has been found to be adaptive: feeding and ingestion rates are higher for bugs in aggregations versus solitary bugs, likely as a result of their feeding strategy (see above). Like many Lygaeidae, *O. fasciatus* must inject seeds with saliva before they are able to feed on them (Schuh and Slater 1995). When many bugs are feeding on the same seed each individual is thought to “economise” on saliva (Root and Chaplin 1976). This is likely to be true for other Lygaeids as other species of the *Oncopeltus* genus have been observed in aggregations containing both adults and nymphs (Root and Chaplin 1976). We will also discuss aggregation in the Lygaeidae further below when we consider the importance of pheromones. While groups are often considered to comprise kin, the extent of nonkin aggregations has yet to be investigated. As mentioned above, in some temperate species adults also aggregate for hibernation. This has been most extensively studied in the species *Lygaeus equestris* where hibernating adults typically enter reproductive diapause before aggregating at overwintering sites.

**Figure 5:**
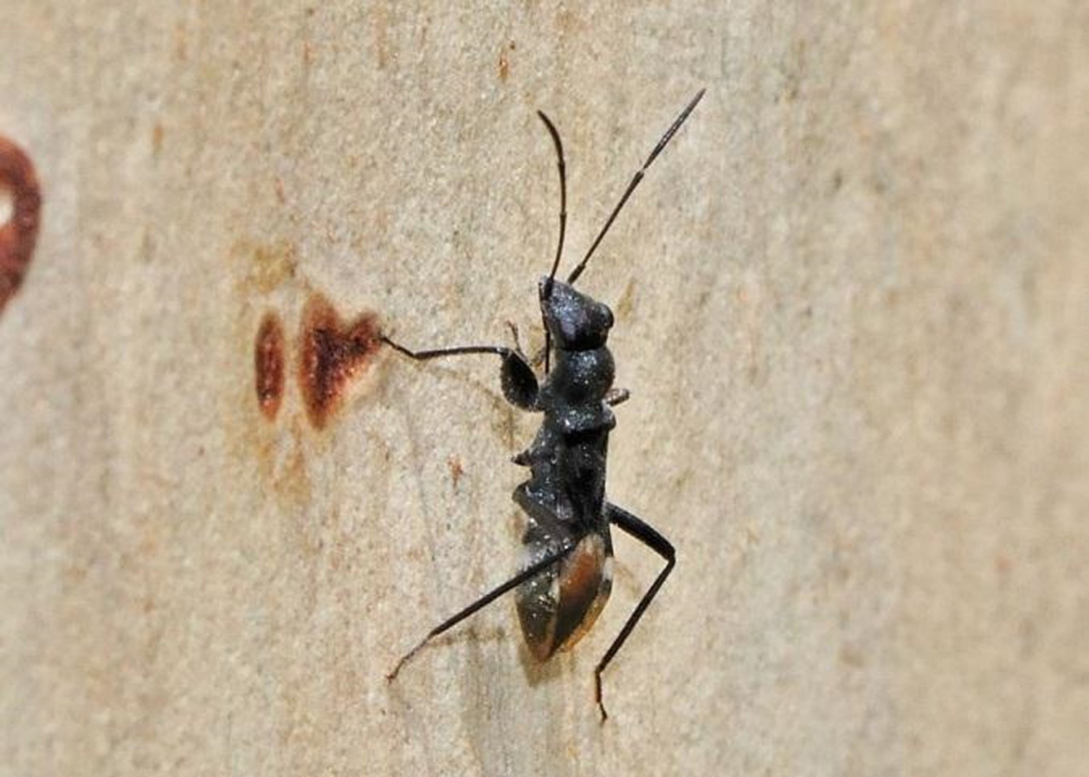
An ant-mimicking Seed Bug - *Daerlac nigricans* – form the Family Rhyparochromidae. Photo courtesy of Peter Chew (http://www.brisbaneinsects.com).

In terms of interspecific interactions, one particularly fascinating interaction concerns ant mimicry or “myrmecomorphy” (McIver and Stonedahl 1993), which is widespread in the Lygaeoidea and appears to have evolved multiple times (Schuh and Slater 1995). This mimicry is thought to act as an anti-predator defence as many predators avoid ants (Durkee et al. 2011) and may be morphological (Fig. 5) or behavioral in nature. For example, *Neopamera bilobata*, a Neotropical lygaeid, moves with jerky, ant-like movements when disturbed. This has been described as “action mimicry” and is thought to cause predators to hesitate in their attack, allowing the bugs time to escape (Schuh and Slater 1995). While some species of Lygaeidae live in or associated with ants nests, it is currently unclear if any species mimic ants for this purpose though (Schuh and Slater 1995).

### Predators and parasites

Despite the interest in the aposematic species of Lygaeidae (see below), there have been few detailed studies of predation in the wild. Generally, it is thought that lygaeids are preyed upon by a variety of predators including birds (Gamberale-Stille and Sillen-Tullberg 1999; Svadova et al. 2010), rodents (Aldrich 1988), and invertebrate predators such as mantids (Berenbaum and Miliczky 1984), while most studies on warning coloration and chemical defences have used model predators such as chicks (Gamberale-Stille and Sillen-Tullberg 1999; but see Berenbaum and Miliczky 1984). In addition, seed bugs have been found in the stomach contents of invasive *Anolis sagrei* in Taiwan and ants have been observed preying upon eggs of *L. equestris* (Kugelberg 1977). However, it seems likely that parasites rather than predators pose the greatest threat to wild Lygaeidae (e.g., Tachinid flies: Lampman and Fashing 1978; Müller and Fritsche 1993; and Trypanosomes: Tieszen et al. 1986; Thorpe and Harrington 1979) especially egg parasites (Root and Chaplin 1976; Lampman and Fashing 1978; Anderson and Solbreck 1992). The impact of parasites appears to vary considerably between populations, for example Solbreck et al. (1989) suggest there is no predation or parasitism in Swedish populations of *L. equestris*, compared with populations in Sicily which fall prey to Tachinid flies (Solbreck et al. 1989).

## Aposematism: Predator Defence and Life History Effects

Much of the research into the evolutionary ecology of the Lygaeidae can be broadly classified into several topics. The first of these is research into the aposematic signals that characterize the best-known species of this group. A related topic is chemical communication, which is central to much of our understanding of lygaeid behavior. We will then cover what is known so far about the population structure of the better-characterized lygaeid species and how this may impact their ecology and evolution. Finally we will consider reproductive behavior, with an emphasis on mating systems and sexual selection in these species, and also the growing area of reproductive interference. Firstly, however, we will consider aposematism.

One of the most striking features of the Lygaeidae is that a number of species, in particular those in the subfamily Lygaeidae, exhibit distinctive black and red patterning (Fig. 3). This is a form of aposematism, whereby the striking colors serve as a warning to predators that the organism possesses anti-predator defences, including toxic chemical defences (Sillen-Tullberg 1985; Ruxton et al. 2004; Mappes et al. 2005). A recent survey of museum specimens at the Natural History Museum in London estimated that more than 20% of the 1951 species of Lygaeidae included have aposematic coloration (Burdfield-Steel, unpublished data). Additionally, 65 (approximately 14%) of the 452 genera surveyed contained at least one aposematic species. Of course, aposematic signals may take forms other than color, such as odor or behavior. Any cue that conveys to the predator that the prey is unprofitable may be considered aposematic. These signals are most effective if they are easily detected (i.e., conspicuous) and memorable, as this facilitates predator detection and learning (Mappes et al. 2005). In the case of the Lygaeidae, this defence is chemical (Aldrich 1988; Aldrich et al. 1997; Zhang and Aldrich 2003). These chemicals are typically cardiac glycosides, also referred to as cardenolides, which are distasteful to most predators (Voneuw et al. 1971; Duffey and Scudder 1972). The prevalence of aposematism within the subfamily Lygaeinae has been linked to their feeding habits. While many lygaeids feed on fallen seeds, members of the Lygaeinae also feed on developing seeds and thus expose themselves to visually oriented predators such as birds (Aldrich 1988). It is hypothesized that this has contributed to the evolution of the bright warning colors so often seen in members of this subfamily (Aldrich et al. 1997).

The availability of toxic compounds that can be sequestered in their food is also important (but also see below). Species such as *S. pandurus*, *O. fasciatus* and members of the genus *Lygaeus* feed on asclepiadaceous plants, such as milkweed, and both adult and larval stages sequester toxic cardiac glycosides obtained from these host plants (Aldrich 1988; Aldrich et al. 1997; Elbanna et al. 2009). Cardiac glycosides inhibit the function of intracellular sodium pumps by binding to the alpha subunit of the sodium (Na+) and potassium (K+) ATPase. As the transportation of Na+ and K+ are crucial for many cellular functions, including muscle contraction, cardiac glycosides are highly toxic to most herbivores (Zhen et al. 2012). The Lygaeidae, in common with other insect species that feed on asclepiadaceous plants, possess amino acid changes in this ATPase that reduce the inhibitory effects of cardiac glycosides. In *O. fasciatus* these chemicals are sequestered in a double layered epidermis and can therefore be released through weak points in the cuticle if the bug is squeezed (Aldrich 1988). The presence of these cardiac glycosides in the bodies of lygaeids makes them unpalatable to both vertebrate predators such as birds (Sillen-Tullberg et al. 1982; Gamberale-Stille and Sillen-Tullberg 1999) and invertebrates. For instance, two species of praying mantis (*Tenodera ardifolia sinensis* and *Mantis religiosa*) have been shown to learn to avoid the bugs. The mantids frequently regurgitated after feeding on milkweed-fed *O. fasciatus* adults and learned to avoid the bugs after only a few encounters (Berenbaum and Miliczky 1984). Additionally, secretions from the scent glands of adult *S. pandurus* contain cardiac glycosides and have been shown to repel birds, cats and even scorpions (Sweet 2000).

In species such as *L. equestris*, *L. simulans*, *L. creticus*, *L. kalmii*, *O. fasciatus* and *S. pandurus*, both adults and nymphs are aposematic and often aggregate, possibly in order to amplify their warning color signals (Sauer and Feir 1973). In an experimental study, the attack rate of domestic chicks on nymphs was found to decrease when nymphs were presented in groups compared with when presented alone (Sillen-Tullberg et al. 2000), suggesting that aggregation may help present a stronger aposematic signal. This suggests a possible link between the evolution of aposematism in Lygaeidae and various life history traits, as aggregation frequency is variable across different species within the (super-) family (Sillen-Tullberg et al. 2000). This would be in keeping with the finding that aposematism is associated with the evolution of gregariousness in butterfly larvae (Sillen-Tullberg 1988). However, in recent experiments we found no effect of chemical defence on aggregation across four different species of lygaeid (Burdfield-Steel et al. 2013). This suggests that while aggregation and aposematism may be linked, the specific level of defence a given individual has may not influence aggregation behavior (i.e., individuals aggregate regardless of their own chemistry). Thus, processes such as automimicry, where individuals perform aggregation behavior despite considerable variation in their own level of chemical defence (Speed et al. 2012), may play a key role, especially in species that can feed on more than one host plant such that levels of defence are likely to fluctuate both within and between individuals.

While much work on aposematism in Lygaeidae has focused on the learning ability of predators rather than the consequences for the bugs themselves, a series of studies by Sillen-Tullberg and colleagues have investigated the effect of diet on aposematic traits. In particular, they found that toxic defences are not always dependent on diet. While toxic defence was found to be food dependent in the polyphagous *L. equestris*, with individuals of the species showing, perhaps unsurprisingly, greater protection from predators (i.e., *Gallus gallus domesticus* chicks) when raised on the toxic *Vincetoxicum hirundinaria* than on the nontoxic *Helianthus annuus*, the naturally monophagous species *Tropidothorax leucopterus* showed no effect of host plant. Thus, *T. leucopterus* was equally unappealing to predators when raised on *H. annuus* (sunflower) as it was when raised on *V. hirundinaria*. Interestingly, however, both species were still relatively unappealing to predators compared with mealworms, suggesting the existence of further defences that are independent of diet. It has been hypothesized that these food-independent defences may be volatile substances or contact poisons produced in the stink glands and released when the animal is startled (Sillen-Tullberg et al. 2000). *O. fasciatus* raised on *H. annuus* have also been found to synthesize a histamine or a histamine-analog (Graham and Staddon 1974), although predation studies using mantids found that sunflower-fed bugs were far less effective at inducing avoidance compared with those fed on milkweed (Berenbaum and Miliczky 1984). This suggests that while some lygaeids suffer a cost of expanding their niche to include nontoxic host plants, others many have developed alternative defences in addition to plant-sequestered toxins, although these appear to provide reduced levels of protection (Sillen-Tullberg et al. 2000). Additionally, as introduced above, lygaeids feeding on nontoxic host plants may benefit from their resemblance to their protected conspecifics feeding on other plant species through automimicry. It has been shown that, after attempting to eat toxic *O. fasciatus,* mantids of the species *Tenodera ardinfolia sinensi* avoided all *O. fasciatus*, even those raised on nontoxic hosts (Berenbaum and Miliczky 1984). This is in keeping with our findings that aggregation behavior in several lygaeid species is independent of individual levels of chemical protection.

There is also evidence that chemical protection can be passed from parents to eggs. The eggs of many lygaeid species, such as *L. equestris*, *S. pandurus* and *O. fasciatus*, are brightly colored, turning from pale yellow through to orange and red as they develop. Recent work in *O. fasciatus* has found that cardiac glycosides are transferred to eggs by both sexes though the maternal contribution is considerably bigger than the paternal (Newcombe 2013; Newcombe et al. 2013). Eggs were found to contain a greater concentration of cardiac glycosides and showed greater protection from a predator (the larvae of the green lacewing, *Chrysopa carnea*) when the mother had been fed on milkweed compared with those where the mother had been feed on sunflower seeds (Newcombe 2013). It is worth noting that these defences were only detected by the lacewing larvae after feeding on, and thus destroying, an egg. Therefore, these defences may benefit close kin by deterring predators from continuing to feed on a clutch.

In summary, the literature surrounding aposematism in insects makes it clear that a level of gregariousness is often associated with aposematic signaling, although much remains to be done to understand how individual levels of defence influence aggregation behavior, including the extent to which animals in the wild are typically (well) defended. For instance, if sequestering and maintaining toxic compounds in the body is energetically costly, is there the potential for “cheats” to evolve that avoid toxic foods but benefit from the mimicry ring of which they are part? It would be interesting to compare oligo- and polyphagous species in this regard, as there may be greater scope for cheating in the latter. As the aposematic lygaeids are often also part of wider interspecific Müllerian mimicry rings (Svadova et al. 2010), there is the potential for complex, spatially variable relationships between intra- and interspecific mimicry, combining Batesian, Müllerian and automimicry. There are clear opportunities to extend our understanding of mimicry in both the laboratory and more importantly in the field with these species. However, now we will move on to focus on the role of chemical communication beyond aposematism, in terms of both the maintenance of sociality in the Lygaeidae, and other aspects of their behavior.

### Pheromones in Lygaeidae – aggregation and communication

One of the suggested mechanisms by which Lygaeidae aggregate is through the use of pheromones; the majority of Heteroptera possess scent glands and they have been found to produce a variety of pheromones (see Table 2) (Aldrich 1988) most of which appear to be for attracting conspecifics, although some may be for defence. For instance, it has been shown that aggregation in nymphs and young adults of *O. fasciatus* and *L. kalmia* is facilitated by the presence of a so-called aggregation pheromone found in acetone extracts taken from the insects (Aller and Caldwell 1979).

**Table 2:** Compounds detected in the pheromones of several species of Lygaeidae

Species	Compounds from Metathoracic scent gland	Defence substances	References
*Lygaeus kalmii*	(E)-2-Hexenyl acetate, (E,E)-2,4-Hexadienyl acetate, (E)-2,5-Hexadienyl acetate, (E)-2-Heptenyl acetate, (E)-2-Octenyl acetate, (E)-2,7-Octadienyl acetate, (E)-2-Hexenyl butyrate, (E,E)-2,4-Octadienyl acetate, (E)-2-Hexen-1-ol, (E)-2-Hexenal, (E)-2-Octenal, (E)-4-oxo-2-Hexenal, (E)-4-oxo-2-Octenal		Aldrich et al. (1999)
*Oncopeltus cingulifer*	(E)-2-Hexenyl acetate, (E,E)-2,4-Hexadienyl acetate, (E)-2,5-Hexadienyl acetate, (E)-2-Heptenyl acetate, (E)-2-Octenyl acetate, (E,Z)-2,6-Octadienyl acetate, (E,E)-2,6-Octadienyl acetate		(Aldrich et al. 1999)
*Oncopeltus fasciatus*	(E)-2-Hexenyl acetate, (E,E)-2,4-Hexadienyl acetate, (E)-2,5-Hexadienyl acetate, (E)-2-Heptenyl acetate, (E)-2-Octenyl acetate, (E)-2,7-Octadienyl acetate, (E,Z)-2,6-Octadienyl acetate, (E,E)-2,6-Octadienyl acetate, (E)-2-Hexenal, (E,E)-2,4-Hexadienal, (E)-2-Octenal, (E)-2,7-Octadienal, (E,Z)-2,6-Octadienal, (E,E)-2,6-Octadienal, 2-Octenal	2-Isobutyl-3-methoxypyrazine	Aldrich et al. (1999, 1997), Games and Staddon (1973)
*Oncopeltus unifasciatellus*	(E)-2-Hexenyl acetate, (E,E)-2,4-Hexadienyl acetate, (E)-2,5-Hexadienyl acetate, (E)-2-Heptenyl acetate, (E)-2-Octenyl acetate, (E)-2,7-Octadienyl acetate, (E,Z)-2,6-Octadienyl acetate, (E,E)-2,6-Octadienyl acetate, (E)-2-Hexenal, (E,E)-2,4-Hexadienal, (E)-2-Octenal, (E)-2,7-Octadienal, (E,Z)-2,6-Octadienal, (E,E)-2,6-Octadienal,		(Aldrich et al. 1999)
*Spilostethus rivularis*	(E)-2-Octenyl acetate, (E)-2-Hexenyl acetate, 3-Methylbutyl acetate, 3-Methyl-2-butenyl acetate, 2-Phenylethanol acetate, (E,E)-2,4-Hexadienyl acetate		(Staddon et al. 1985)
*Geocoris punctipes*	(E)-2-Octenyl acetate, (E)-2-Hexenyl acetate, (E)-2-Octenal, (E)-2-Hexenal, (E)-4-oxo-2-Hexenal, (E)-2-Decenal		Marques et al. (2000)
*Geocoris varius*	(E)-2-Hexenal, (E)-2-Decenal, Tridecane		Yamashita and Kanehisa (1979)
*Neacoryphus bicrucis*	(E,E)-2,4-Hexadienyl acetate, (E)-2-Octenyl acetate, 2-Phenylethanol acetate, (E)-2-Hexenal, (E)-2-Octenal, (E)-4-oxo-2-Hexenal, (E)-4-oxo-2-Octenal, (E,E)-2,4-Hexadienyl acetate, 2-Phenylethanol acetate		Aldrich et al. (1999, 1997)
*Oxycarenus hyalinipennis*	(Z,E)-3,7,11-Trimethyl-1,3,6,10-dodecatetraene, (E)-2-Octenyl acetate, (E)-2-Octenal, 2,6,6-Trimethylbicyclo[3.1.1]hept-2-ene, 1-Methyl-4-(1-methylethenyl)-cyclohexene, 2-Hexenal, 1,3,3-Trimethyl-2-oxabicyclo[2.2.2.]octane, (E)-2-Hexenyl acetate, 2-Octenal, (E)-4-oxo-2-Hexenal, 2-Octenyl acetate, (E)-4-oxo-2-Octenal		Knight et al. (1984), Olagbemiro and Staddon (1983)
*Tropidothorax cruciger*	(E)-2,7-Octadienyl acetate, (E)-2-Octenyl acetate		Aldrich et al. (1997)

Like other Heteroptera, lygaeids possess both metathoracic scent glands (MTG) and dorsal abdominal scent glands (DAG; Aldrich 1988). As discussed above, the secretions of both have been found to contain cardenolides sequestered from the plants they feed on. These odors are thought to act as an additional aposematic signal in their own right to deter potential predators (Aldrich et al. 1997) as many Insectivora, which are important predators of litter-dwelling Lygaeidae, are color-blind (Aldrich 1988). Distinctive scents could therefore act in the same way as distinctive markings and coloration to ward off potential visual predators such as birds.

In several species, the MTG is reduced in size (Aldrich 1988); however, the chemistry of the MTG is complex and has been found to be sexually dimorphic in *Oncopeltus fasciatus* (Aldrich 1988) and *Spilostethus rivularis* (Staddon et al. 1985) among others. The presence of sexually dimorphic pheromones in adult lygaeids suggests they may play a role in mate choice. Males of *Neacoryphus bicrucis* and *Tropidothorax cruciger* have been found to produce compounds that attracted adults of both sexes in field bioassays (reviewed by Millar 2005). Similarly, a study of *O. fasciatus* and *L. kalmii* by Aldrich et al. (1999) found that pheromone traps baited with synthetic pheromone blends mimicking those extracted from the metathoracic scent glands of both species attracted females of the corresponding species. As both *O. fasciatus* and *L. kalmii* often have patchy distributions, it has been suggested that males which colonize new patches of host plants use long-range pheromones to guide potential mates to the patch. In addition, conspecific males and nymphs were also attracted to such pheromones (Aldrich et al. 1999). As such, these signals may indicate the presence of food and hence suitable habitat. Finally, females of the predatory species *Geocoris punctipes* produce pheromones that increase activity, specifically searching behavior, in males (Millar 2005). Clearly then, pheromonal signals must be considered when seeking to understand both migration dynamics and mate acquisition in these bugs.

We may expect signals such as pheromones to change over an individual's lifetime (e.g., virgin versus mated, diapause versus reproductively active). Indeed, the cotton seed bug *Oxycarenus hyalinipennis* changes the compounds synthesized by the MTG after the first day of adult life. It seems likely that the differences in scent glands often found between nymphs and adults (Aller and Caldwell 1979; Schuh and Slater 1995) may be reflective of the different roles pheromones play at these life stages, but data remain limited to date.

Another key chemical signaling system in insects is that associated with their cuticular hydrocarbon (CHC) repertoire. CHCs are long-chain fatty acids which are secreted onto the cuticle from specialized epidermal cells. These waxy compounds function primarily as protection from desiccation (i.e., water-proofing). However, the interspecific diversity of CHCs presented on the cuticle is also used by a wide variety of species for species-discrimination (Kather and Martin 2012), and sex-specific differences in CHC blends are also used for sex-discrimination and in processes such as mate choice (Everaerts et al. 2010; Thomas and Simmons 2010). Finally, there is growing evidence that CHCs also allow discrimination of self from nonself (e.g., in the cricket *Gryllodes sigillatus*, females appear to recognize males they have already mated with by the presence of their own CHCs left on the male during previous copulations: (Weddle et al. 2012)). Despite this broad literature across a wide-range of insects, there is surprisingly little information about CHCs in the Lygaeidae (e.g., Jackson 1983). We have recently begun to address this deficit, and have explored CHC complements in five species (*Lygaeus equestris, L. creticus, L. simulans, Spilostethus pandurus and Oncopeltus fasciatus*). We have shown CHC variation across species, across the sexes within the species, and some evidence for differences associated with developmental stage (Burdfield-Steel, Smith and Shuker, unpublished). This work suggests that cuticular hydrocarbons in lygaeids may have similar chemical communication roles as in other invertebrates, and be involved in similar processes such as mate choice and reproductive isolation.

### Population structure and ecology – Migration, diapause and genetic structuring

Despite the interest in terms of pest status and studies of migration, there have been very few genetic studies of the population structure of lygaeid species. To date, a study of allozyme frequencies among populations of *Lygaeus equestris* in Sweden has revealed evidence of genetic differentiation between the populations (Sillen-Tullberg 1983), genetic differentiation that was echoed in differences in physiological response to photoperiod across the Swedish populations. A study of laboratory-reared specimens taken from four different locations in Sweden revealed a latitudinal cline in critical photoperiod, with longer critical photoperiods in the north and shorter photoperiods required to trigger reproductive diapause in southern populations (Solbreck and Sillen-Tullberg 1981). Similarly, populations of *O. fasciatus* in different regions have also been found to be genetically distinct and show variation in reproductive diapause and migration tendency between populations (Dingle 1972; Dingle et al. 1980a,b1980b). While *O. fasciatus* have not been found to overwinter in specific sites in the same way as *L. equestris*, some populations do show delayed reproduction and migration. Individuals from populations in variable, temperate climates can be triggered to enter diapause far more easily than those from more stable, tropical climates (Dingle et al. 1980a). This fits with the generally accepted theory that populations in the north of the species range are seasonal migrants that move north from subtropical overwintering sites during the spring (Chaplin and Chaplin 1981). Further support for this comes from studies showing that bugs from these more northern populations have increased flying ability (Dingle et al. 1980b). *Spilostethus pandurus* also shows reproductive diapause triggered by falling temperatures. For instance, in northern India they pass the winter hibernating in leaf litter and fewer overlapping generations were found than in the south (Sweet 2000). Whether the recorded differences in life history and host plant between populations in different geographic locations is reflected by genetic structuring in this species remains unknown though.

Another form within- and among-population variation can take is in terms of wing polymorphism, as lygaeids show a great deal of variation both in flying ability and wing morphology (Slater 1977; Solbreck and Anderson 1989). Currently, studies across many insects groups suggest that wing polymorphisms, such as brachyptery (described below), may be the result of trade-offs between flying ability and fecundity (Roff 1986). There are many degrees of wing modifications within the Heteroptera, though only a few are typically found within the Lygaeidae. These fall into a four distinct categories (Schuh and Slater 1995): (1) aptery, or the complete absence of wings; (2) sub-brachyptery where the forewings only reach to the end of the firth abdominal tergite; (3) brachyptery where the forewings are reduced and do not cover the sixth and seventh abdominal terga and the hind wings are reduced but usually not flap-like; (4) macroptery where the clavus and corium are distinct, the membrane is well-developed and the hind wings are elongate.

Natural populations of *Nysius huttoni* have been found to contain three wing forms. Field surveys over a four year period found that approximately 94.1% of the population were macropters, 5.5% were sub-brachypters and 0.4% were brachypters. Additionally, photoperiod was found to affect the production of the different wing morphs in the laboratory, with long photoperiods favoring the production of macropters. This was suggested to be a mechanism to enable the rapid dispersal of adults to new host plants during dry summers, which may reduce the availability of the bug's preferred weedy hosts (Wei 2011). In the genus *Oncopeltus*, species in isolated habitats have been found to display both aptery and brachyptery, presumably as a result of selection for reduced dispersal. Additionally, flying ability within species can show considerable variation, even without obvious changes to wing morphology (Dingle et al. 1980b). When *Oncopeltus fasciatus* from both migratory and nonmigratory populations were subjected to artificial selection for wing length in the laboratory, both showed a positive response, and flying ability also responded positively to selection (Dingle and Evans 1987; Palmer and Dingle 1989). It has been observed that within the Lygaeinae, wing reductions typically occur in ground-living species that feed upon fallen seeds while those that feed upon plants typically retain their flying ability. This may be the result of the increased habitat complexity experienced by these latter species, necessitating greater mobility (Solbreck et al. 1990). Wing polymorphisms in Lygaeidae have also been linked to habitat permanency though. Slater (1977) observed that ground-living Lygaeidae in old, stable areas such as southwest Australia and the Cape of South Africa have a greater degree of wing modification and are more likely to have flightless morphs compared with those in less stable areas (Slater 1977). Interestingly, size and wing morphology seem to be correlated with wing dimorphisms, with aptery occurring more frequently in smaller species, although the reason for this pattern remains unknown (Solbreck et al. 1990).

As life history traits such as diapause and migratory capabilities have been shown to vary in several species, and that this variation between populations has been shown to remain despite laboratory culture (Solbreck and Sillen-Tullberg 1981), it is reasonable to assume that many Lygaeidae species have structured populations and that this structure has allowed the evolution of climate-specific behavioral and physiological adaptations (i.e., local adaptation). This has been supported by work by Dingle and colleagues in *O fasciatus* which found variation in many traits including diapause (Dingle et al. 1980a), body size and flight ability (Dingle et al. 1980b) between tropical, subtropical and temperate populations. Additionally differences in body size and sexual harassment costs have been found in lab populations of *L. equestris* collected from different locations in Europe (Shuker et al. 2006). The lack of recent genetic studies does, however, make it harder to predict how fine a scale this structuring extends. Many species, including *L. equestris* (Solbreck and Sillen-Tullberg 1990) and *O. fasciatus* (Aldrich et al. 1999) are limited to a few plant species and as a result often have a patchy distribution across large parts of their ranges. For example, mark and recapture studies carried out in Sweden in the 1970s found that in areas of patchy food-plant distribution 82% of *L. equestris* individuals marked remained within the same area, no more than 50 meters from where they were originally captured, even after migration to overwintering sites (Sillen-Tullberg 1983). While this seems initially like a high level of isolation, the inferred migration rate of 0.18 would still be sufficient to limit genetic differentiation and without studies using markers such as microsatellites or single-nucleotide polymorphisms (SNPs), capable of detecting relatively recent genetic differentiation, it is impossible to know if such spatial structuring of the bugs’ habitat is reflected in patterns of gene flow. At least one closely related species pair found in Europe, *L. equestris* and *L. simulans*, are hypothesized to have arisen via some form of local adaptation, possibly associated with pre- or postglacial isolation (Hewitt 1999, 2001).

More generally, one area that has received rather little attention in the Lygaeidae is that of speciation. As mentioned above, *L. simulans* and *L. equestris* may represent a relatively recent speciation event, perhaps associated with repeated range changes across Europe associated with glaciation events. Despite asymmetric reproductive isolation between the two; male *L. simulans* can mate successfully with *L. equestris* females, but *L. equestris* males are usually unable to fertilize *L. simulans* females (Evans 2011), it is unknown whether introgression and/or reinforcement played any role in the evolution of reproductive isolation between these sibling species. Classic patterns of speciation, such as Haldane's Rule (Coyne and Orr 2004), also remain to be tested. Hybridization has also been found in several species from the *Oncopeltus* genus (O'Rourke 1979; Leslie and Dingle 1983). Barriers to hybridization are frequently asymmetric within this genus as well and, despite the occasional observations of hybrid pairs in the wild, laboratory experiments confirmed strong conspecific mating preferences (Leslie and Dingle 1983).

### Mating systems – promiscuity, sperm competition and harassment

From the species studied to date, lygaeids seem to be characterized by promiscuous mating systems with both males and females mating multiple times (Wang and Davis 2006). For instance, mating frequencies in both wild and laboratory populations of *Neacoryphus bicrucis* have been observed to average 0.8 copulations per day for females (McLain 1989) and in laboratory populations of *O. fasciatus* 20–30% of individuals within a colony may be engaged in copulation at any one time (Economopoulos and Gordon 1972). Preliminary courtship behaviors (or at least those that are easily observable) are very rare but exceptions do exist, for example in *N. huttoni* males have been observed to court females by antennating them prior to mounting (Yang and Wang 2004), though how typical this behavior is remains unknown. Fertilization is internal, and males produce free spermatozoa rather than spermatophores (Dallai and Afzelius 1980). In the majority of species described, copulation is initiated by the male grasping the female with all three pairs of his legs. Males then orient themselves in the same direction as the female to initiate genital coupling. If genital coupling is successful, males then change orientation and the majority of the mating takes place with males and females facing opposite directions (i.e., back-to-back). This copulation behavior appears to be the norm within the family and has been recorded in a number of species including *L. equestris* (Sillen-Tullberg 1981; Shuker et al. 2006) (Fig. 4), *S. pandurus*, *O. fasciatus* (Walker 1979) and *Nysius huttoni* (Yang and Wang 2004).

Encounter polygynandry appears to be the predominant mating system within the group with little evidence of either resource-based (e.g., resource defence) or nonresource-based territoriality (e.g., lekking). There is some evidence that male Lygaeidae attract potential mates via pheromonal cues (see above; (Aldrich et al. 1999; Zhang and Aldrich 2003) or exclude other insects from food patches (McLain and Shure 1987), but whether these behaviors are sufficient to be considered different from encounter polygynandry is unclear. In particular, the exclusion of other individuals from food patches by males of species such as *N. bicrucis* appears to be a side effect of indiscriminate sexual harassment rather than directed behaviors toward potential rivals (but see Rodriguez 2000). This behavior may also result in the exclusion of female conspecifics, reducing the future mating opportunities of the males in those patches (McLain and Pratt 1999).

As with many insect species (Garcia-Barros 2000; Salavert et al. 2011; Kanuch et al. 2013) increased female body size in the Lygaeidae is typically correlated with higher fecundity (but see Shuker et al. 2006). As a result we expect males to preferentially mate with larger females and indeed weak selection for larger females has been detected in laboratory populations (Dougherty and Shuker 2014). Despite this, evidence of precopulatory selection in the Lygaeidae is generally rather scarce, although some patterns of nonrandom mating have been detected (*Neacoryphus bicrucis*, larger males gain more matings, McLain 1992; in *Nysius huttoni*, males prefer females with broader abdomens, Yang and Wang 2004). Within *L. equestris*, for example, there is suggestion that females prefer smaller, or intermediate sized males (this despite previous findings suggesting females gain fitness benefits from mating with heavier males, see below) (Dougherty and Shuker 2014). Chemical protection does not influence precopulatory mate choice in this species either (Burdfield-Steel et al. 2013), despite the suggestion that such protection may be passed on to offspring (Newcombe 2013).

**Figure 6:**
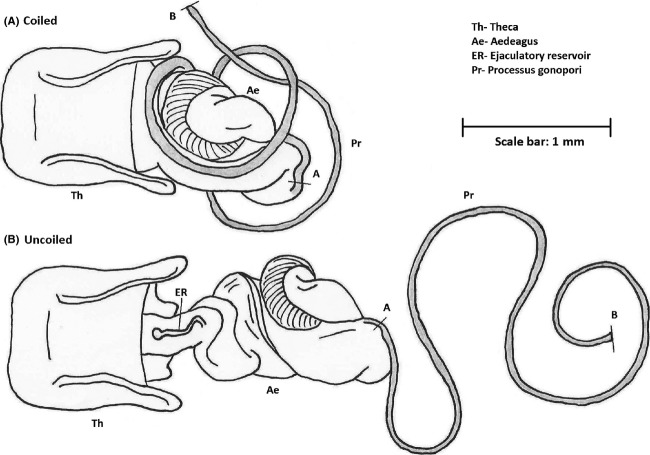
Diagram of adegus of a male *Lygaeus equestris*. Courtesy of Liam Dougherty.

Females commonly mate with one or several males between oviposition events (Economopoulos and Gordon 1972; Sillen-Tullberg 1981; Wang and Davis 2006). This creates the potential for intense sperm competition (e.g., Simmons 2001) and studies *of L. equestris* have shown that the last male to mate with a female before oviposition fertilizes approximately 90% of the eggs she lays in that batch (Sillen-Tullberg 1981). Similarly, studies in laboratory strains of *O. fasciatus* found that when two males were allowed to mate with a single female in succession, the mean number of progeny fathered by the first male is only 14% of the mean number of progeny of the second male (Economopoulos and Gordon 1972), although additional research suggested this ratio can vary according to the photoperiod during which the second mating takes place (Walker 1979). As such, changes in both male and female (physiological) state can perhaps significantly alter insemination and fertilization success. Overall, patterns of nonrandom fertilization success may well be related to the complexities of lygaeid genitalia (see Fig. 6). As in common within the Heteroptera, copulations are often prolonged (Sillen-Tullberg 1981; although insemination may not be) and depending on the species copulation duration can vary from less than an hour to over 15 h. As successful insemination has been found to occur in matings less than an hour long, these extended copulations have been suggested to be a form of mate-guarding (Sillen-Tullberg 1981; Wang et al. 2008). This hypothesis was supported by a study of *N. bicrucis* in 1989 which found that the mean fertilization success of the last male to mate with a female before oviposition was 78% despite a single insemination containing enough sperm to fertilize 10–20 clutches of eggs (McLain 1989). This study also found that not only did females frequently re-mate before oviposition following matings of short (i.e., less than 8 h) duration, but that longer matings were more frequent when the sex ratio was male biased. The latter observation suggests plasticity of copulatory behavior, both in terms of copulatory mate-guarding and also perhaps in terms of strategic ejaculate allocation (i.e., longer copulations and greater sperm transfer with increasing sperm competition intensity: Parker and Pizzari 2010; Kelly and Jennions 2011), and similar plasticity has been found in *N. huttoni* (Wang et al. 2008). Interestingly, however, longer copulations may not always result in greater sperm transfer as findings in *L. equestris* suggest that longer copulation durations produced no increase in the number of fertilized eggs, and the insemination rate was highest during the first hour of copulation (Sillen-Tullberg 1981). The results of a recent study manipulating sex ratio suggests that while male *L. equestris* respond to male-biased sex ratios by increasing copulation duration, they do not reduce it in response to female-biased sex ratios (for more on the effect of sex ratio on male behavior see Wang et al. 2009). Indeed no significant difference was detected in average copulation duration when a male was housed with a single female verses four females (Burdfield-Steel et al., in review). Thus, there may be limits to the plasticity of these behaviors.

The genitalia of both male and female lygaeids are typically complex (Bonhag and Wick 1953) and elongated (for details of the genetic patterning of these genitalia see Aspiras and Angelini 2011; Aspiras et al. 2011). During mating males must thread their tube-like intromittent organ (or processus gonopori) through the female's spermathecal complex, in order to deliver their flagellated sperm to the receptaculum and thus successfully inseminate her (Tadler 1999). Since not all matings result in insemination and fertilization, it is possible that cryptic female choice plays a role in the overall process of sexual selection in these species (Eberhard 1996). However, this has yet to be confirmed and the exact mechanism of any possible form of cryptic choice is still uncertain. Gschwentner and Tadler (2000) suggested that a valve structure at the entrance to the female's spermatheca could allow females to deny entry to both the male organ and sperm; however, a subsequent study utilizing three (albeit different) species, *O. fasciatus*, *Leptoglossus occidentalis* and *Leptocoris trivittatus* (Chiang 2010), has suggested that the male organ instead gains access to the spermatheca duct via a parallel duct named the insemination duct, thus bypassing the valve. However, as this was discovered in the box elder bug, *L. trivittatus*, it is uncertain whether it holds true for all Lygaeidae. Intriguingly, despite the male intromittent organ often exceeding half his body length (Higgins et al. 2009) there appears to be no positive selection for increased length. Indeed, studies in *L. equestris* and *L*. *simulans* have found evidence for stabilizing or negative selection acting on male intromittent organ length (Tadler 1999; Higgins 2009) although this may vary depending upon the fitness measure used. It appears that in *L. equestris*, males with longer genitalia are less likely to gain matings. However, when fertilization success is taken into account selection on genitalia is stabilizing (Dougherty and Shuker 2014). This suggests that different selective pressures may act on male genitalia at different stages of reproduction.

The lack of strong precopulatory selection, combined with the complex genitalia and high rate of variability in fertilization success, suggest that postcopulatory choice plays a significant role in the sexual selection of the Lygaeidae. In their 2006 study, Shuker et al. found that when female *L. equestris* were allowed to mate only once, those that mated with heavier males produced a greater number of eggs. This suggests that females may benefit from matings with some males more than others. It is possible then that females may have responded to the apparent male control of copulation by evolving mechanisms whereby they can bias paternity following the initiation of copulation. However, experimental evidence of postcopulatory selection in the Lygaeidae is scarce. While it has been hypothesized that behaviors such as the rocking and kicking that can occur during copulation may be attempts by one of both of the sexes to influence paternity, experimental studies have failed to detect a measurable effect of these behaviors on fertilization success (Sillen-Tullberg 1985). This is despite kicking behavior in females being found to reduce copulation duration (Rodríguez 1998). Thus, the prevalence of postcopulatory selection within the Lygaeidae remains an interesting avenue for further study.

With so few courtship preliminaries, males effectively harass females as they try and engage genitalia. Studies have demonstrated a fitness cost to females of this repeated mating and harassment, confirming the existence of sexual conflict over mating frequency in Lygaeidae (Shuker et al. 2006). Sexual conflict, whereby the optimal strategies of males and females do not align during reproduction, has been recognized as a driving force in the evolution of reproductive morphologies and behavior (Parker 1984; Eberhard 1996; Arnqvist and Rowe 2005). A key source of sexual conflict within the Lygaeidae appears to be mating frequency, with the optimal number of matings for males far exceeding that of females. Females often appear to resist male attempts to mate (Rodriguez 2000) and males may continue to attempt to mate with “unreceptive” females for several hours (pers. obs.). Females may well then have to balance the costs of a suboptimal mating rate with those of resisting male mating attempts. Studies in *L. equestris,* for example*,* have demonstrated a significant reduction in female longevity when females are housed with multiple males compared within one or no males (Shuker et al. 2006). This reduction was greatly reduced when males were rendered unable to mate, suggesting that while harassment by males may impose some costs the females, it is mating itself which is responsible for most of the costs observed (perhaps through damage imposed by the internal movements of the complex genitalia). Further evidence for a cost of mating in Lygaeidae comes from a study of the seed bug *Togo hemipterus*, which found that mating reduces lifespan and starvation tolerance in both males and females (Himuro and Fujisaki 2010). Intriguingly, this species has also been found to produce seminal proteins that inhibit female remating (Himuro and Fujisaki 2008), opening up the possibility for similar seminal-fluid mediated sexual conflicts over mating and reproduction as to those seen in *Drosophila* (e.g., Chapman et al. 1995).

Finally, sexual behavior needs not always be present. Remarkably, a unisexual population of the seed bug *Nysius groenlandicus* has been found in Northern Greenland. In some areas of the Zackenberg Valley within the Northeast Greenland National Park, there exist populations in which males are rare or entirely absent. Due to the persistence of these unisexual populations it is assumed that females in these areas reproduce parthenogenetically. Unusually these asexual populations occur in close proximity to sexual populations (Bocher and Nachman 2011). *N. groenlandicus* is widespread across northern Europe and is common throughout Greenland. It is a well-adapted arctic species and is univoltine, with all reproductive activity taking place during the brief arctic summer and only the eggs surviving the winter. The preferred habitat of the species is dry, sunny areas with sparse vegetation and a temperature of 30°C. As a result it has a patchy distribution and its obligatory univoltinism makes it vulnerable to local extinction during particularly bad summers. It is thought that parthenogenetic reproduction may provide an advantage in such adverse conditions as it removes the need for females to find a mate prior to egg-laying, thus reducing the time needed to successfully complete an annual reproduction cycle. This is supported by the finding that differing sex ratios can be explained by climatic factors, with the female bias increasing with distance from the coast. As inland climatic conditions are more predictable than those along the coast it would seem that asexual reproduction provides a benefit in relatively stable environments while sexual reproduction remains the preferred mode of reproduction in the more changeable coastal regions (Bocher and Nachman 2010). However, it is also possible that these asexual populations are the result of a male-killing parasite, like those described earlier, rather than an adaptation.

### Reproductive interference – ecological and evolutionary consequences

As is assumed in many species, it is thought that male seed bugs can increase their fitness by mating with as many females as possible (Bateman 1948). This selection for males to mate readily means that in areas where related species coexist, there is the potential for interspecific matings to occur. Interspecific matings and attempted matings have been observed in several species of Lygaeidae, both in the wild (Leslie and Dingle 1983; McLain and Pratt 1999) and in the laboratory (D. M. Shuker, N. Currie, T. Hoole, and E. R. Burdfield-Steel, *in prep*). These interspecific mating interactions have been shown to have significant impacts both at an individual (Shuker et al., *in prep*) and an ecological scale (McLain and Shure 1987; McLain and Pratt 1999), and as such are examples of reproductive interference (Groning and Hochkirch 2008; Burdfield-Steel and Shuker 2011). On an individual scale, harassment and mating by interspecific males have been shown to impose a fitness cost on female *L. equestris* (Shuker et al., *in prep*). Large decreases in both longevity and fecundity, comparable with those experienced in the presence of a conspecific male, were recorded in females housed with male *S. pandurus*. No such costs were recorded when *L. equestris* females were housed with females of either species. A similar pattern was found in *N. bicrucis* (McLain and Pratt 1999). Females housed with males of either the same species or *Margus obscurator* following mating with a conspecific male had approximately half the fecundity of those housed with females of either species. On an ecological scale, this cost of heterospecific courtship and harassment has been suggested as a reason why the two species are rarely found coexisting on the same plant despite having overlapping ranges (McLain and Pratt 1999). In addition, a series of studies carried out on *N. bicrucis* in the field suggest that indiscriminate, and often aggressive, mating behavior carried out by males of the species actively excludes other polyphagous insect species from habitats supporting high *N*. *bicrucis* densities (McLain and Shure 1987). Thus, there is emerging evidence that reproductive interference may play a significant role in determining habitat use in the Lygaeidae.

## Conclusion

The Lygaeidae offer a variety of opportunities for evolutionary and behavioral ecologists, and we hope this review will make the super-family better known and encourage more groups to begin working with them. They are often easy to keep in the laboratory, and in the age of next-generation sequencing and subthousand dollar genomes, any species can relatively easily become a fully fledged genetic model organism (for an example of this see Zhen et al. 2012). While our own interests focus on sexual selection, sexual conflict, and reproductive interference, we would also like to emphasize: (1) the fascinating pattern of coevolution between the bugs and their endosymbionts, including the evolution of specialized structures and the remarkable diversity *within genera*; (2) the opportunities to explore individual variation in chemical defence and how this variation might influence within- and among-population and species patterns of mimicry; (3) the patterns of cannibalism that remain poorly studied, including egg- and sib-cannibalism, and the opportunities for testing kin selection theory; (4) the lack of precopulatory mate choice and the likely importance of postcopulatory sexual selection; and (5) the ease with which these species can be used to study fundamental aspects of life history evolution, including diet specialization, wing polymorphisms, and diapause.
